# The National Tobacco Bill

**Published:** 1899-04-08

**Authors:** 


### The Hospital

A Journal of

The Medical Sciences and Hospital Administration.

Vol. XXVI.—No. 654.] EDITED BY SIR HENRY BURDETT, K.C.B., AND SOLOMON C. SMITH, M.D., M.R.C.P. [April 8, 1899.

### THE NATIONAL TOBACCO BILL.

It was once said by a jubilant Chancellor of the Exchequer that the people of Great Britain had speedily drank themselves out of a debt by which any other country would have been seriously and for a long time embarrassed; and his successor of to-day is probably equally jubilant at the discovery that the national income has much exceeded the estimate on which the Budget of last year was founded. There can be no doubt that the drink bill, as it is the fashion of total abstainers to call it, even if it has increased in amount, has been more evenly distributed over the population than was the case in former years, and that rational use and enjoyment have come to bear a constantly increasing proportion to excess. It would be interesting to know what is the present amount of the national tobacco bill, at what rate it is increasing, and what effects can be legitimately attributed to its increase. According to the last returns, the Customs revenue from tobacco and snuff—the latter being probably now very small in proportion to the former—amounted in round figures to eleven millions and a-half sterling, or, also roughly, to six shillings and six-pence a head yearly for every man, woman, and child in the United Kingdom. As the duty bears a large proportion to the value, the actual expenditure of consumers might probably be set at about fifteen millions; and it is worth while to consider how much of this represents absolute waste, mere sensual indulgence of a fleeting character, and how much of it represents the supply of a want, or confers upon the nervous system of the consumer any increase of power to grapple with the facts of life. That it does this, more or less, is loudly claimed for tobacco by its votaries; and it is difficult, if not impossible, to test the validity of the claim by experiment, or to obtain any trustworthy evidence upon the question. The mere statement of the consumer goes for little, because it may well happen that he fails to estimate his own powers correctly, either when stimulated by tobacco or when deprived of it. The judicial bench in a past generation was adorned by a learned person who was wont to say of himself that, when sober, he was so merry, boyish, hilarious, as to be unfit for the transaction of serious business; "but," he added, "when I am drunk I am as sober as a judge." It is not recorded that suitors and counsel were always prepared to agree with him in his opinion.

The only information approaching to evidence which we possess cannot be described as unanimously favourable to tobacco. The authorities of two American universities are said to have classified the students into smokers and non-smokers, and to have found that the former beat the latter in everything, alike in the schools and in the gymnasium. Medical and legal teachers in this country would probably declare that the most hopeless of all individuals, the "chronic" student, lives habitually in a fool's paradise engendered of tobacco, and that he is always confident of passing his examination on the next occasion, without the preliminary process of working for it. We have never met with a wastrel or an idler who did not smoke; and every physician is familiar with examples of the injury done by tobacco taken in excess, a word which can only be defined with reference to the idiosyncrasy of the individual. On the other hand, it is at least probable that the evil consequences of the drug, whatever they may be, are produced with greater certainty upon the immature nervous system than upon that of the adult; for thousands of men of middle age smoke regularly without the smallest manifest impairment either of the *mens sana* or of the *corpus sanum*. We have sometimes wondered, nevertheless, whether the annual consumption of fifteen millions worth of tobacco may not be exerting a slowly prejudicial influence upon the national character. It was said of a living statesman by one of his admirers, "What I like about him is his you-be-damnedness"; and "you-be-damnedness," at least, in the sense of "Oh, it is all right," appears to be one of the influences under which British commerce is showing a disposition to languish, and to make way for competitors who regard strenuousness as the high road to success. The *Times* paper of March 31st contained an exceedingly interesting and important letter from Mr. Philip E. Pilditch, apparently a builder in large business, who described the way in which he was forced, against his will, to obtain his materials, such as girders, slates, and other things, from American or Continental sources of supply, and who gave a graphic but melancholy account of the tranquil and ignorant indifference of the English dealer, or the English commercial traveller, as contrasted with the complete knowledge, the promptitude, and the desire to meet the views and wishes of a customer which were displayed by his foreign rivals. A gentleman who represented a British firm of slate dealers "had never seen the quarries whose products he was trying to sell, could not tell if a thinner slate could be supplied at a lower price, gently deprecated any inquiry on the subject being made at the quarry, and was unaware that American or German slates were being offered in the market at much lower price than British." "He was," continues Mr. Pilditch, "quite a representative British traveller"; and we should not be surprised if he were also a great authority upon the brands of cigars. Neither should we be surprised if the thousands of young men engaged in trade, who, in a street or on a railway platform, are never without pipes in their mouths, come in time to merit a similar description.

